# Prevalence of genetically defined familial hypercholesterolemia and the impact on acute myocardial infarction in Taiwanese population: A hospital-based study

**DOI:** 10.3389/fcvm.2022.994662

**Published:** 2022-09-12

**Authors:** Yen-Ju Chen, I-Chieh Chen, Yi-Ming Chen, Tzu-Hung Hsiao, Chia-Yi Wei, Han-Ni Chuang, Wei-Wen Lin, Ching-Heng Lin

**Affiliations:** ^1^Department of Medical Research, Taichung Veterans General Hospital, Taichung, Taiwan; ^2^Division of Allergy, Immunology and Rheumatology, Department of Internal Medicine, Taichung Veterans General Hospital, Taichung, Taiwan; ^3^Institute of Clinical Medicine, National Yang Ming Chiao Tung University, Taipei, Taiwan; ^4^Institute of Biomedical Science and Rong Hsing Research Center for Translational Medicine, National Chung Hsing University, Taichung, Taiwan; ^5^School of Medicine, National Yang Ming Chiao Tung University, Taipei, Taiwan; ^6^Department of Public Health, College of Medicine, Fu Jen Catholic University, New Taipei City, Taiwan; ^7^Institute of Genomics and Bioinformatics, National Chung Hsing University, Taichung, Taiwan; ^8^Cardiovascular Center, Taichung Veterans General Hospital, Taichung, Taiwan; ^9^Division of Cardiology, Department of Internal Medicine, Taichung Veterans General Hospital, Puli Branch, Nantou, Taiwan; ^10^Department of Life Science, Tunghai University, Taichung, Taiwan; ^11^Department of Health Care Management, National Taipei University of Nursing and Health Sciences, Taipei, Taiwan; ^12^Department of Industrial Engineering and Enterprise Information, Tunghai University, Taichung, Taiwan; ^13^Institute of Public Health and Community Medicine Research Center, National Yang Ming Chiao Tung University, Taipei, Taiwan; ^14^Department of Medical Research, China Medical University Hospital, Taichung, Taiwan

**Keywords:** familial hypercholesterolemia, prevalence, CAD, AMI, LDLR polymorphisms

## Abstract

**Background:**

Familial hypercholesterolemia (FH) is a common genetic disorder with markedly increased risk of coronary artery diseases (CAD), especially acute myocardial infarction (AMI). However, genetic tests for FH are not always necessary in the current diagnostic criteria of FH, which might lead to underestimation of the prevalence of FH and a lack of awareness of FH-associated CAD and AMI. We aimed to explore the prevalence of genetically defined FH in the hospital-based population and to determine the impact of FH risk variants on CAD and AMI.

**Methods:**

The study participants were recruited between June 24, 2019 and May 12, 2021, at a medical center in Taiwan, in cooperation with the Taiwan Precision Medicine Initiative (TPMI) project. The prevalence of FH was calculated and the effects of FH pathogenic variants on CAD and AMI were analyzed by logistic regression models and shown as ORs and 95% CI.

**Results:**

The prevalence of genetically defined FH was 1.13% in the hospital-based population in Taiwan. Highest LDL and total cholesterol levels were observed in patients with *LDLR* rs28942084 (LDL 219.4±55.2; total cholesterol 295.8±55.4). There was an approximately 4-fold increased risk of hyperlipidemia in subjects with the *LDLR* rs769446356 polymorphism (OR, 4.42; 95% CI, 1.92-10.19) and AMI in individuals with the *LDLR* rs730882109 polymorphism (OR, 3.79; 95% CI, 2.26-6.35), and a 2-fold increased risk of CAD in those with the *LDLR* rs749038326 polymorphism (OR, 2.14; 95% CI, 1.31-3.50), compared with the groups without pathogenic variants of FH.

**Conclusions:**

The prevalence of genetically defined FH was 1.13% in the hospital-based population in Taiwan, which was higher than the rate observed in individuals with clinically defined FH. The risk of CAD and AMI was increased to varying degrees in subjects with different FH risk alleles. Close monitoring and risk stratification strategy are essential in high-risk patients with FH risk alleles to facilitate early detection and treatments.

## Introduction

Familial hypercholesterolemia (FH) is a common autosomal-dominant inherited disorder characterized by an elevated low-density lipoprotein (LDL) cholesterol level. A markedly higher risks of cardiovascular diseases in subjects with FH were reported previously, with a 13-fold increased risk of coronary artery diseases (CAD), a 2-fold increased risk of incident acute myocardial infarction (AMI), and a 1.28- to 2.5-fold increased risk of recurrent AMI (1, 2). The genetic pathogenesis of FH includes pathogenic variants in low-density lipoprotein receptor (LDLR), apolipoprotein B (APOB), and proprotein convertase subtilisin/kexin type 9 (PCSK9) genes, which result in impaired function of LDL receptor, defects in APOB causing impaired binding with the LDL receptor, and gain-of-function mutations in PCSK9 causing LDL receptor degradation, respectively. There are heterozygous and homozygous forms of FH: heterozygous FH is the most common monogenic disorder and is associated with early atherosclerotic coronary heart disease owing to elevated LDL level >160 mg/dL if untreated in children and >190 mg/dL if untreated in adults. Homozygous FH is a rare but more severe form of FH associated with premature mortality before 30 years old if left untreated. A 4-year-old child with homozygous FH who died of acute myocardial infarction (AMI) with near-complete occlusion of the coronary artery has been reported previously (3).

Cardiovascular mortality increases in FH subjects if untreated, ranging from approximately 100-fold greater mortality in young adults aged 20-39 to 4-fold greater mortality in adults in middle age (40–59 years), compared with the general population (4). Of note, AMI accounts for one of the major causes of cardiovascular death in FH patients and 50% of men and 15% of women with heterozygous FH will die of AMI before middle age if untreated (5). Nowadays, HMG-CoA (3-hydroxy-3-methyl- glutaryl-coenzyme A) reductase inhibitors (statins) are the mainstream treatments for FH, and the cardiovascular mortality rate decreases significantly after using statins, with 1 mmole/L LDL reduction resulting in a 20% reduction in major vascular events in a prospective meta-analysis of 14 randomized trials of statins (6), and a reduction of about one-third in prospective studies in the United Kingdom and the Netherlands (4, 7). There was also a 23% reduction in myocardial infarction or coronary death, and a 24% reduction in the need for coronary revascularization (6) after widespread use of statins.

A number of diagnostic criteria have been developed for FH to enhance early diagnosis and early treatment, such as the US Make Early Diagnoses Prevent Early Deaths Program Diagnostic Criteria (US MEDPED), the Simon Broome criteria, the Dutch Lipid Clinic Network (DLCN) criteria, and the Taiwan FH diagnostic criteria (8–10). However, the current diagnostic criteria comprise phenotype and genotype characteristics, with phenotypes predominating. Genetic tests for FH are not always necessary in all diagnostic criteria and have not been widely implemented in clinical practice. A recent meta-analysis revealed the prevalence of FH was 1:311 subjects (11), but it might have been underestimated because non-universal FH genetic tests were used and therefore potential candidates for FH may not have been noticed. In 2018, The Journal of American College of Cardiology (JACC) experts released a consensus suggesting FH genetic tests for FH cases and at-risk individuals (12). With the widespread implementation of FH genetic tests, early detection of FH pathogenic variants and early treatments are becoming increasingly important in efforts to reduce FH-associated mortality and morbidity, especially major cardiovascular diseases (5).

For early intervention by risk stratification strategy, it is necessary to develop risk assessment tools for CAD and AMI risk prediction in FH patients. The traditional CAD risk scores, such as the Framingham score, are not practical in risk prediction due to the early onset of CAD in FH cases. The Montreal-FH-SCORE was thus developed for this group of patients (AUC 0.840, 95% CI 0.808–0.872, *p* < 0.0001). It is composed of simple clinical variables, and there is a 10.3-fold higher risk of CAD events in FH patients with hyperlipidemia if the score is high (above 20) compared to that of patients with a low score (95% CI 6.7–15.7, *P* < 0.0001) (13). However, it is currently unclear whether the Montreal-FH-SCORE is applicable to FH patients without the development of hyperlipidemia. Polygenic risk scores have been proposed for earlier risk prediction of CAD in FH cases (14, 15), especially for those with negative standard FH genetic test, but it is not suitable for subjects with a positive FH genetic test and includes some single nucleotide polymorphisms (SNPs) with a very low minor allele frequency, which might reduce the power. CAD risk assessments in patients with monogenic FH were reported, but the subdivided effects of single allele and the risk prediction for major adverse cardiovascular events, such as AMI, still lack evidence (16, 17). Therefore, the frequency of FH in the general population based on genotype screening, for both definite FH cases and at-risk individuals, is currently unclear, and it has yet to be established to what extent different FH risk alleles contribute to increased CAD and AMI risks in FH patients. This study aimed to explore the prevalence of FH in the hospital-based population using genotyping assessment, to determine the impact of FH variants on CAD and AMI, and to develop a risk stratification for genetically identified FH subjects.

## Materials and methods

### Enrollment of participants and identification of AMI cases

From June 24, 2019 until May 12, 2021, we recruited patients aged ≥ 20 years at a medical center in Taiwan, in cooperation with the Taiwan Precision Medicine Initiative (TPMI) project, which is overseen by Academia Sinica, Taiwan. The following subjects were excluded: patients with leukemia in the active stage, patients who a received blood transfusion in the past 6 months, and patients with malignancy receiving chemotherapy or radiotherapy within 1 year. Detailed information on participants was collected using medical records and blood tests, and all participants were genotyped with an Affymetrix TWB 2.0 SNP chip, containing three FH risk genes: *LDLR, APOB*, and *PCSK9*. There were seven susceptibility alleles in *LDLR* (rs730882109, rs769446356, rs749038326, rs28942084, rs875989921, rs121908029, rs761954844), two susceptibility alleles in *APOB* (rs144467873, rs5742904), and one allele in *PCSK9* (rs28942111) on the Affymetrix TWB 2.0 SNP chip, all of which were hotspots in Taiwan's population based on information released by Taiwan's biobank (TaiwanView: http://taiwanview.twbiobank.org.tw). Subjects with at least one susceptibility allele associated with FH were considered to be genetically defined FH patients. Non-FH controls were those without FH alleles, matching FH cases for age and gender with a ratio of 1:10. All of the participants provided informed consent and the study was approved by the ethics committee of Taichung Veterans General Hospital's Institutional Review Board (IRB No. SF19153A). This study was followed the Strengthening the Reporting of Observational Studies in Epidemiology (STROBE) reporting guidelines.

### Genotyping and quality controls

The blood DNA samples were obtained from the participants and were genotyped using the Axiom Genome-Wide TWB 2.0 Array Plate (Affymetrix, Santa Clara, CA, USA) (18). The Affymetrix TWB 2.0 SNP chip contains 653,291 SNPs and was designed specifically for Taiwan's Han Chinese population. Affymetrix Power Tools was used for analysis and a quality control procedure was performed to exclude markers that failed Hardy-Weinberg equilibrium tests with *P* < 1.0 × 10–5, minor allele frequency <0.05, and genotype missing rate of > 5%. A total of 591,048 SNPs were retained after the quality control.

### Data collection and identification of covariates

Covariates including baseline characteristics, selected comorbidities, and biochemical data were collected. Comorbidities were identified by ICD-9 codes, including hyperlipidemia (ICD-9 code 272), hypertension (ICD-9 code 401–405), diabetes mellitus (ICD-9 code 250), chronic kidney disease (CKD, ICD-9 code 580–587), AMI (ICD-9 code 410), CAD (ICD-9 code 411–413, 414.00, 414.01), and cerebrovascular accident (CVA, ICD-9 code 433–438), if the diagnostic ICD-9 code was documented three times or more during outpatient visits or at least once in a hospitalization. In addition to using ICD-9 codes, hyperlipidemia was also identified if a patient had a low density lipoprotein (LDL) value of more than 130 mg/dL, or was using a lipid-lowering agent.

Biochemical data included lipid profiles (LDL, high density lipoprotein [HDL], triglyceride [TG], and total cholesterol), blood sugar status (fasting glucose level and hemoglobin A1c [HbA1c]), serum uric acid level, renal function (serum creatinine and estimated glomerular filtration rate [eGFR]), and liver enzyme (alanine aminotransferase [ALT], aspartate aminotransferase [AST]). The associations of FH pathogenic variants with hyperlipidemia and cardiovascular diseases were examined by logistic regression.

### Statistical analysis

The demographic information is shown as mean ± standard deviation (SD) for continuous variables and number (percent) for categorical variables. Student's *t*-test for continuous variables and Chi-square test for categorical variables were conducted to compare variables between FH cases and non-FH controls. Fisher's exact test was used to compare variables between LDLR group and APOB group. Odds ratios (OR) and 95% confidence interval (95% CI) of FH pathogenic variants were calculated by multivariate logistic regression analysis to adjust for potential confounders, and the effects of variants on hyperlipidemia and cardiovascular diseases were explored. All data were analyzed using the Statistical Package for the Social Sciences (SPSS) version 23.0. Statistically significance was set at *p*-values < 0.05.

## Results

### Baseline characteristics of the participants

**Figure 1:**
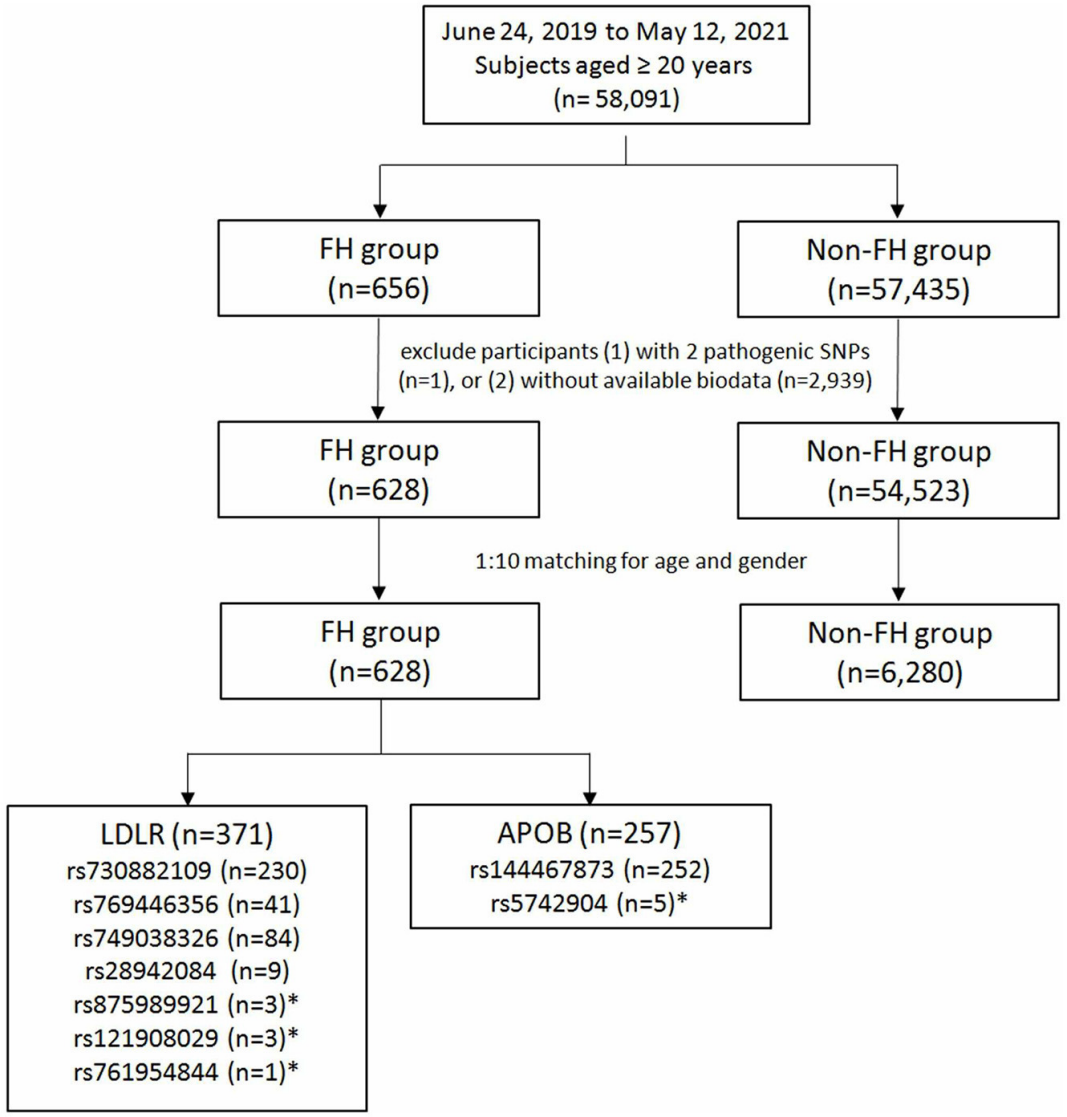
Study participants enrollment flow chart. FH, familial hypercholesterolemia; LDLR, low-density lipoprotein receptor; APOB, apolipoprotein B. *Not included in the final analysis due to limited case numbers.

The flowchart of participant enrollment is shown in Figure 1. In total, 58,091 participants were recruited and genotyped, including 656 (1.13%) with susceptibility alleles associated with FH and 57,435 (98.87%) non-FH controls. The prevalence of familial hyperlipidemia in Taiwan was 1:88 subjects using genotyping assessments. One subject with two FH risk alleles and 2,939 subjects without biodata were excluded. Finally, there are 371 patients with *LDLR* variants, 257 patients with *APOB* variants, but no patients with *PCSK9* variant. There were 628 subjects in the FH group and 6,280 in the control group in the final analysis after matching for age and gender with a ratio of 1:10.

Among the 628 subjects with FH susceptibility alleles, there were 371 (59.08%) participants with *LDLR* and 257 (40.92%) with *APOB*. The allele frequencies were 36.62% in *LDLR* rs730882109, 6.53% in *LDLR* rs769446356, 13.37% in *LDLR* rs749038326, 1.43% in *LDLR* rs28942084, 0.48% in *LDLR* rs875989921, 0.16% in *LDLR* rs761954844, 0.48% in *LDLR* rs121908029, 40.13% in *APOB* rs144467873, and 0.80% in *APOB* rs5742904. In patients with *APOB* polymorphisms, there were two patients with homozygous genotype while the others had heterozygous genotypes. SNPs with lower allele frequencies were excluded in the following analysis.

Table 1 shows participants with FH susceptibility alleles exhibited higher percentages of hyperlipidemia (71.0 vs. 54.5%, *p* < 0.0001), AMI (5.6 vs. 2.3%, *p* < 0.0001), and CAD (26.9 vs. 22.7%, *p* = 0.02). Higher LDL (147.0 ± 49.1 vs. 117.3 ± 35.9 mg/dL, *p* < 0.0001) and total cholesterol (229.6 ± 59.8 vs. 195.0 ± 45.6 mg/dL, *p* < 0.0001) were observed in subjects with FH susceptibility alleles, compared with their counterparts.

**Table 1:** Comparisons of baseline characteristics and comorbidities between FH and non-FH groups.

**Variable**	**FH (*****n*** = **628)**		**non-FH (*****n*** = **6,280)**		***p-*value**
	***n***	**%**	***n***	**%**	
**Age (mean/SD)**	59.3	15.1	59.3	15.1	-
**Gender**					
Female	337	53.7	3370	53.7	-
Male	291	46.3	2910	46.3	
**Comorbidities**					
Hyperlipidemia	446	71.0	3424	54.5	**<0.0001**
Hypertension	241	38.4	2448	39.0	0.77
DM	167	26.6	1805	28.7	0.26
CKD	109	17.4	1272	20.3	**0.08**
AMI	35	5.6	147	2.3	**<0.0001**
CAD	169	26.9	1424	22.7	**0.02**
CVA	115	18.3	1115	17.8	0.73
**Biochemistry (mean/SD)**					
LDL (mg/dL)	147.0	49.1	117.3	35.9	**<0.0001**
HDL (mg/dL)	54.1	17.9	55.0	16.3	0.44
Triglyceride (mg/dL)	136.8	105.5	134.7	95.4	0.68
Total cholesterol (mg/dL)	229.6	59.8	195.0	45.6	**<0.0001**
Uric acid (mg/dL)	6.06	1.8	6.12	1.8	0.54
Fasting glucose (mg/dL)	112.3	40.6	113.0	37.9	0.70
HbA1c (%)	6.5	1.6	6.4	1.7	0.40
eGFR (mL/min/1.73m2)	86.3	26.1	85.1	25.1	0.23
Creatinine (mg/dL)	1.04	1.20	1.03	1.1	0.91
ALT (mg/dL)	32.0	39.9	31.4	50.3	0.77
AST (mg/dL)	30.2	39.2	29.8	41.4	0.87

Categorical variables were expressed as numbers (percent) and were analyzed using the Chi-square test.

Continuous variables were expressed as mean ± standard deviation (SD) and were analyzed using Student's t-test for normal data distributions.

FH, familial hypercholesterolemia; DM, diabetes mellitus; CKD, chronic kidney disease; AMI, acute myocardial infarction; CAD, coronary artery disease; CVA, cerebrovascular disease; LDL, low density lipoprotein; HDL, high density lipoprotein; HbA1c, glycosylated hemoglobin; eGFR, estimated Glomerular filtration rate; ALT, alanine aminotransferase; AST, aspartate transferase.

The bold values indicate the statistically significant values.

### The distribution of cholesterol level among different FH risk alleles

**Figure 2:**
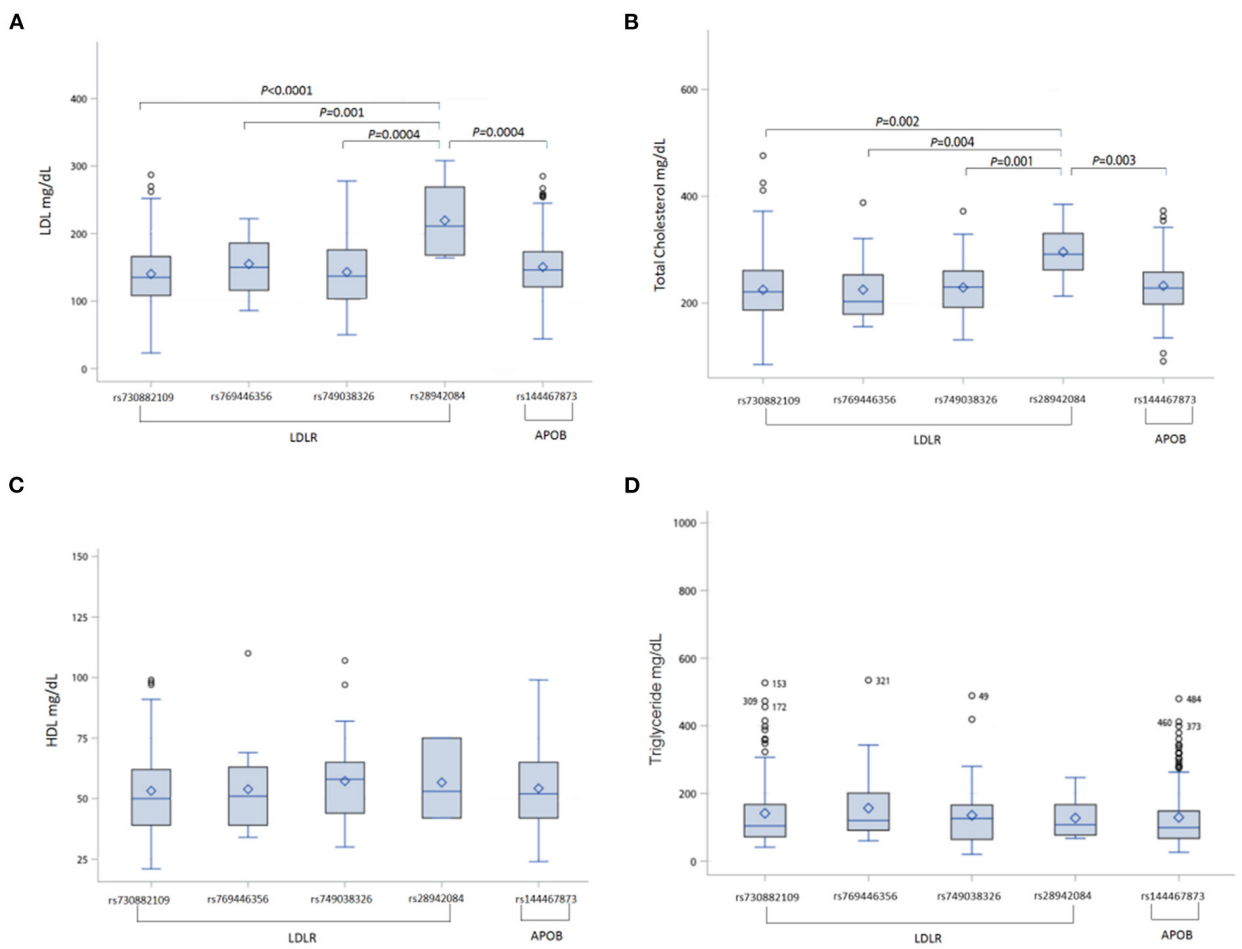
Comparisons of cholesterol and triglyceride levels among each of the FH risk alleles in FH patients **(A)** LDL level, **(B)** total cholesterol level, **(C)** HDL level, **(D)** triglyceride level. FH, familial hypercholesterolemia; LDLR, low-density lipoprotein receptor; APOB, apolipoprotein B; LDL, low density lipoprotein; HDL, high density lipoprotein.

Then, we analyzed the association between FH susceptibility alleles in the FH group and comorbidities, and the characteristics of comorbidities and serology in each of the FH risk alleles are shown in Table 2. Despite, each risk allele in the FH group without statistically significant relationship among the different comorbidities, there were marked differences in serum LDL and total cholesterol levels among the different FH risk alleles, as shown in the Table 2. In additional, he highest LDL and total cholesterol levels were observed in patients with *LDLR* rs28942084 (LDL 219.4 ± 55.2, *p* < 0.001; total cholesterol 295. ± 55.4, *p* = 0.02) (Figures 2A,B). There was no significant variance in serum HDL (Figure 2C) or TG levels (Figure 2D) among the FH risk alleles.

**Table 2:** Comparisons of comorbidities and serology among each of the FH risk alleles in FH patients.

**Variable**	**LDLR (*****n*** = **364)**								**APOB (*****n*** = **252)**		***p-*value**
	**rs730882109** **(*****n*** = **230)**		**rs769446356** **(*****n*** = **41)**		**rs749038326** **(*****n*** = **84)**		**rs28942084** **(*****n*** = **9)**		**rs144467873** **(*****n*** = **252)**		
	***n***	**%**	***n***	**%**	***n***	**%**	***n***	**%**	***n***	**%**	
**Age (mean/SD)**	59.3	14.4	61.3	14.7	59.5	15.9	62.4	9.2	58.9	15.6	0.85
**Gender**											
Female	117	50.9	21	51.2	47	56.0	6	66.7	140	55.6	0.74
Male	113	49.1	20	48.8	37	44.1	3	33.3	112	44.4	
**Comorbidities**											
Hyperlipidemia	164	71.3	33	80.5	57	67.9	7	77.8	178	70.6	0.66
Hypertension	102	44.4	17	41.5	29	34.5	1	11.1	88	34.9	0.08
DM	67	29.1	12	29.3	20	23.8	0	-	65	25.8	0.33
CKD	41	17.8	6	14.6	9	10.7	4	44.4	48	19.1	0.09
AMI	19	8.3	3	7.3	2	2.4	0	-	11	4.4	0.19
CAD	68	29.6	13	31.7	29	34.5	2	22.2	55	21.8	0.13
CVA	44	19.1	10	24.4	13	15.5	3	33.3	43	17.1	0.53
**Biochemistry (mean/SD)**											
LDL	140.3	47.4	154.9	41.4	142.9	51.0	219.4	55.2	150.4	49.0	**<0.001** ^a, b, c, d^
HDL	53.2	19.6	53.9	18.1	57.2	17.2	56.7	16.8	54.2	16.4	0.85
Triglyceride	141.2	114.0	156.6	101.3	135.3	88.0	127.1	62.6	129.1	104.5	0.63
Total cholesterol	225.0	62.3	225.2	58.7	229.2	52.1	295.8	55.4	232.3	59.2	**0.02** ^a, b, c, d^
Uric acid	6.2	2.0	6.0	1.7	5.8	1.6	5.5	1.9	6.0	1.6	0.65
Fasting glucose	115.4	46.7	106.6	29.4	115.4	45.4	96.0	10.9	109.7	35.1	0.53
HbA1c	6.4	1.7	6.6	1.9	6.4	1.3	5.7	0.3	6.5	1.6	0.79
eGFR	86.3	28.7	80.4	16.6	87.7	25.2	82.5	20.8	86.6	25.1	0.64
Creatinine	1.1	1.3	0.9	0.2	1.0	0.9	0.9	0.3	1.0	1.3	0.82
ALT	32.0	32.6	47.0	56.5	32.3	31.5	23.2	15.3	30.2	45.7	0.16
AST	30.5	44.7	38.1	31.6	30.2	22.7	21.3	6.3	29.4	40.7	0.82

FH, familial hypercholesterolemia; LDLR, low-density lipoprotein receptor; APOB, apolipoprotein B; DM, diabetes mellitus; CKD, chronic kidney disease; AMI, acute myocardial infarction; CAD, coronary artery disease; CVA, cerebrovascular disease; LDL, low density lipoprotein; HDL, high density lipoprotein; HbA1c, glycosylated hemoglobin; eGFR, estimated Glomerular filtration rate; ALT, alanine aminotransferase; AST, aspartate transferase.

Post-hoc analysis p < 0.05,

a rs730882109 vs. rs28942084;

b rs769446356 vs. rs28942084;

c rs749038326 vs. rs28942084;

d rs28942084 vs. rs144467873.

### The association of FH polymorphism with hyperlipidemia and cardiovascular diseases

To examine the impact of individual polymorphism associated with FH on hyperlipidemia and cardiovascular diseases, multivariate logistic regression analyses were performed to adjust for potential confounders, as shown in Table 3. Subjects with *LDLR* rs28942084 polymorphism had the highest risk of hyperlipidemia, albeit without statistical significance (OR, 4.83; 95% CI, 0.95–24.62, *p* = 0.06), compared with the non-FH controls (non-FH controls were those without FH alleles). The risk of hyperlipidemia was significantly associated with *LDLR* rs769446356 polymorphism (OR, 4.42; 95% CI, 1.92–10.19, *p* = 0.001), compared with the non-FH controls. Increased risk of AMI was found in subjects with the *LDLR* rs730882109 polymorphism (OR, 3.79; 95% CI, 2.26–6.35, *p* < 0.0001), and *APOB* rs144467873 polymorphism (OR, 2.21; 95% CI, 1.12–4.03, *p* = 0.02). Furthermore, there was higher risk of CAD in patients with *LDLR* rs749038326 (OR, 2.14; 95% CI, 1.31–3.50, *p* = 0.002), and an approximately 4-fold increased risk of AMI in individuals with the *LDLR* rs730882109 polymorphism (OR, 3.79; 95% CI, 2.26–6.35, *p* < 0.0001), compared with the non-FH controls.

**Table 3:** Association of FH risk alleles and comorbidities examined by multivariate logistic regression analyses.

**Variables**	**Hyperlipidemia**			***p-*value**	**AMI**			***p-*value**	**CAD**			***p-*value**
	**OR**	**95% CI**			**OR**	**95% CI**			**OR**	**95% CI**		
LDLR rs730882109 (*n* = 230)	2.43	1.76	3.37	**<0.0001**	3.79	2.26	6.35	**<0.0001**	1.43	1.05	1.94	**0.03**
LDLR rs769446356 (*n* = 41)	4.42	1.92	10.19	**0.001**	3.42	1.00	11.72	0.05	1.56	0.77	3.15	0.22
LDLR rs749038326 (*n* = 84)	2.48	1.49	4.14	**0.001**	1.22	0.29	5.11	0.79	2.14	1.31	3.50	**0.002**
LDLR rs28942084 (*n* = 9)	4.83	0.95	24.62	0.06	-	-	-	-	1.40	0.27	7.27	0.69
APOB rs144467873 (*n* = 252)	2.75	2.02	3.74	**<0.0001**	2.12	1.12	4.03	**0.02**	1.02	0.73	1.40	0.93

Adjusted by age, gender, hypertension, diabetes mellitus, and chronic kidney disease.

FH, familial hypercholesterolemia; LDLR, low-density lipoprotein receptor; APOB, apolipoprotein B; AMI, acute myocardial infarction; CAD, coronary artery disease.

The bold values indicate the statistically significant values.

## Discussion

This study demonstrated the prevalence of FH was 1.13% (one in 88 subjects) in the hospital-based population in Taiwan using comprehensive genotyping screening. Higher LDL and total cholesterol level were observed in the FH groups. There was an approximately 4-fold increased risk of hyperlipidemia in subjects with *LDLR* rs769446356 and AMI in individuals with the *LDLR* rs730882109 polymorphism, and a 2-fold increased risk of CAD in those with *LDLR* the rs749038326 polymorphism, compared with the groups without pathogenic variants of FH.

We established the prevalence of FH in Taiwan via genotyping screening in the general population, and the rate was higher than in previous reports. Early reports revealed the prevalence of FH was around 1:500 individuals in limited cases, but more recent studies suggested that the prevalence of FH might be twice that figure or higher, according to larger studies using a systemic approach (5, 19). Due to the concern of founder effects which bias the data toward a greater than expected prevalence, Hu et al. (11) reported the prevalence of FH was 1:311 subjects according to the results of a meta-analysis of 62 studies after excluding founder populations. However, because the previously available data were evaluated mainly based on family studies, small-scale hospital cases, or selected populations, the actual prevalence of FH worldwide might still be underestimated (20). In addition, the available data on the FH diagnostic rate ranged from 71% in the Netherlands to <1% in most countries/territories worldwide. The mean global diagnostic rate of FH was shown to be <1% and was extremely underdiagnosed (5). With comprehensive genetic screening, regardless of whether phenotypes have developed, we found a higher prevalence of FH in the general population in Taiwan, where no obvious founder effects were reported previously (21). Although partly because the study conducted in a hospital-based population, our result was similar to the findings of a study which enrolled whites of Danish descent, an unselected community-based population comprising 69,016 individuals with heterozygous FH. The prevalence of FH was 0.73% (one in 137) in the Danish general population. The analysis combined definite and probable FH diagnosed by DLCN criteria and general genotyping (22). The results are encouraging as they indicate that genetic information itself could suggest the possibility of FH index cases before symptoms or signs develop, making primary preventive strategy and early intervention in at-risk individuals achievable.

In the present study, our results indicate that there is a clear need to implement an FH genetic screening program. Indeed, some patients with FH could be overlooked since CAD, such as AMI, is the leading cause of mortality worldwide, and more common risk factors for CAD might mask the impact of genetically elevated LDL levels (23). The prevalence rates of traditional CAD risk factors were markedly higher than those of FH in patients with AMI. For this reason, physicians might not be sufficiently alert to the possibility of FH, and FH cases were often diagnosed after the development of AMI and serious adverse cardiovascular events. Li JJ and colleagues reported that 3.5% of patients undergoing coronary angiography were ultimately found to be cases with definite/probable FH phenotypes (24). Brown et al. surveyed 135 individuals who visited the Advanced Lipids Disorders Clinic at Johns Hopkins Hospital, and 21% of patients with hyperlipidemia had FH as determined by genetic testing (25). Incorporating genetic tests identified an additional 10.4% (14 in 135 subjects) of FH patients who did not meet the LCN, Simon Broome, or MEDPED criteria. Therefore, the application of FH genetic tests in clinical practice could help early detection of FH cases and guide risk stratification programs for FH intervention at an individual level (26). The Familial Hypercholesterolemia Foundation convened the international JACC expert consensus panel in 2018 and recommended FH genetic tests, which detect the *LDLR, APOB*, and *PCSK9* genes, as the standard of care for FH. Some rationales were released, including improving earlier and definitive diagnosis of FH, and identifying FH patients with higher cardiovascular risks who needed more aggressive lipid lowering plans with better adherence (12).

Despite the heterogeneous genetics of FH and the different detection rates of numerous genetic screening methods, our results are consistent with previously reported data. Huang CC and his colleagues determined the spectrum of FH genetic mutations in Taiwan in clinically diagnosed FH patients based on hyperlipidemia (LDL >190 mg/dL) and in those meeting the Taiwan FH diagnostic criteria (27). By using custom-made mass spectrometry, targeted next generation sequencing, or multiplex ligation-dependent probe amplification, the results revealed the distribution of FH variants in 445 patients in Taiwan was as follows: 86.6% *LDLR* mutations, 12.7% *APOB* mutations, and 0.7% *ABCG5* mutations. No *PCSK9* mutation was detected. *APOB* rs144467873 was the most common variant (12.6%), followed by *LDLR* rs761954844 (11.5%) and *LDLR* rs730882109 (10.8%). These reported proportions of FH risk genes were similar to those found in our study, although the *LDLR* rs761954844 polymorphism was rare in our analysis of the general population, regardless of whether the phenotypes were present or not. The association of *LDLR* rs761954844 and the development of symptoms/signs warrants further study.

Genetic mutation status could predict AMI risks independently (HR [hazard ratio] 4.51, 95% CI 1.74–11.7) (26) and Ron Do et al. demonstrated that rare mutations in *LDLR* accounted for 0.24% of variants for AMI, of which 49% was associated with FH (28). In the present study, we found an approximately 4-fold increased risk of AMI in patients with *LDLR* rs730882109 polymorphism, and a 2-fold increased risk in those with the *APOB* rs144467873 polymorphism. The overall incidence of AMI in FH and non-FH group was 5.6 and 2.3%, respectively. It should be noted that because no patients with *LDLR* rs28942084 polymorphism developed AMI in our cohort during the registry period, there were no data on the associations of AMI and *LDLR* rs28942084, which was the variant with the highest level of LDL and total cholesterol. Nevertheless, close monitoring of serum lipid profile and early intervention with statins should be considered in this group of patients with the *LDLR* rs28942084 polymorphism since a causal relationship between genetically increased lipoprotein (a) levels and risk of AMI has been documented previously (29). Further genetic research on the association of the *LDLR* rs28942084 polymorphism and risk of AMI is warranted. These findings indicate that preventive strategies need to be designed based on risk stratification of patients with different polymorphisms, especially as FH-associated AMI and adverse cardiovascular events in affected subjects are preventable with early detection and treatments (4).

This study has several strengths. First, this study was a population-based investigation of the impact of different FH risk alleles on hyperlipidemia and cardiovascular diseases with a large sample size, which allowed enough power for analysis. In addition, we used the Axiom Genome-Wide TWB 2.0 Array Plate, which is a validated chip with tight quality control and it performed well in terms of the similarity of its results to those reported in another study in Taiwan using distinct methodology (27). The exploration of the effect of tailor-made hotspot genes was more feasible in clinical practice.

However, there were some limitations in our study. First, the participants were all East Asians, and thus, the results may not be generalizable to Western populations. Further genetic studies on different ethnicities and subgroups are warranted. Second, our results are not applicable to polygenic hypercholesterolemia patients, estimated to be nearly 20% of clinically defined FH patients (30). However, as the majority of FH the patients had monogenic hypercholesterolemia, we can still apply our findings to the general population when it comes to designing a risk stratification strategy. Moreover, further study on polygenic FH is needed to shed light on this issue. Third, physical details such as tendon xanthomata or arcus cornealis, which might be present in patients with FH, as well as lifestyle risk factors of CAD, such as smoking and alcohol, were not recorded in the study. Therefore, we could not completely exclude the potential confounders in the present study, which should be explored in future studies.

This study sheds new light on the exact prevalence of genetically defined FH in the hospital-based population, and may be helpful in developing risk stratification strategy recommendations based on FH risk variants. To identify patients with FH as early as possible is essential in terms of decreasing FH-associated morbidity and mortality, as well as reducing social burdens. Based on the results of this study, the authors recommend the following. In light of the efficacy of FH genetic screening, the broad implementation of FH screening programs should be considered, which may provide considerable beneficial effects on health, which in turn are likely to have a positive impact on society and the economy. Furthermore, to reduce FH-associated morbidities and cardiovascular mortality, we suggest that patients with *LDLR* rs28942084 polymorphism should be closely monitored to track serum LDL and total cholesterol levels, since a causal relationship between increased lipoprotein levels and risk of AMI has been proven. For patients with *LDLR* rs730882109, *LDLR* rs749038326, and *APOB* rs144467873 polymorphisms, we recommend aggressive correction of risk factors for CAD, including hypertension as well as insulin intolerance, and early prevention of the occurrence of CAD, especially AMI events, by conducting regular electrocardiograms, exercise stress tests, and so on.

## Conclusion

The prevalence of genetically defined FH was 1.13% in the hospital-based population in Taiwan, which was higher than the clinically defined rate of FH reported previously. The risk of hyperlipidemia and AMI was markedly increased by approximately 4-fold in subjects with the LDLR rs769446356 or LDLR rs730882109 polymorphisms, and the risk of CAD increased significantly by 2-fold in those with the LDLR rs749038326 polymorphism. Patients with the LDLR rs28942084 polymorphism developed the highest LDL and total cholesterol level. Therefore, widespread implementation of screening programs with FH genetic tests should be considered, followed by close monitoring, and risk stratification strategy in high-risk patients with specific FH risk alleles to reduce FH-associated morbidities and cardiovascular mortality.

## Data availability statement

The data used in this study cannot be made available in the manuscript, the supplemental files, or in a public repository due to the Personal Information Protection Act executed by Taiwan's government, starting in 2012. The data presented in this study are available on request from the corresponding author.

## Ethics statement

The studies involving human participants were reviewed and approved by the Ethics Committee of Taichung Veterans General Hospital Institutional Review Board (IRB No. SF19153A). The patients/participants provided their written informed consent to participate in this study.

## Author contributions

Y-JC and C-HL conceptualized the study. Y-JC, I-CC, Y-MC, and C-HL were responsible for data curation. C-YW and I-CC were responsible for formal analysis. Y-MC and C-HL were responsible for funding acquisition. T-HH, H-NC, and W-WL were responsible for investigation. Y-MC, T-HH, H-NC, C-HL, and W-WL were responsible for methodology and were responsible for the resources. T-HH, H-NC, C-HL, and W-WL provided supervision. Y-JC, I-CC, Y-MC, C-YW, and C-HL were responsible for the validation. Y-JC, I-CC, and Y-MC were responsible for visualization and wrote the original draft. Y-JC, I-CC, W-WL, and C-HL reviewed and edited the manuscript. All authors contributed to the article and approved the submitted version.

## Funding

This study was funded by a grant from Taichung Veterans General Hospital Research Fund (Registration number TCVGH-YM1100103, TCVGH-YM1110104, TCVGH-1107303C, TCVGH-1117311C, and TCVGH- 111G211), Taichung Veterans General Hospital/National Health Research Institutes Joint Research Program (grant number TCVGH-NHRI11001 and TCVGHNHRI11101), and Academia Sinica (grant number Academia Sinica 40-05-GMM and AS-GC-110-MD02). The funder was not involved in the study design, collection, analysis or interpretation of data, the writing of this article, or the decision to submit it for publication.

## Conflict of interest

The authors declare that the research was conducted in the absence of any commercial or financial relationships that could be construed as a potential conflict of interest.

## Publisher's note

All claims expressed in this article are solely those of the authors and do not necessarily represent those of their affiliated organizations, or those of the publisher, the editors and the reviewers. Any product that may be evaluated in this article, or claim that may be made by its manufacturer, is not guaranteed or endorsed by the publisher.
